# Ethics of overtreatment and undertreatment in older adults with cancer

**DOI:** 10.1186/s12910-025-01255-9

**Published:** 2025-07-24

**Authors:** Clark DuMontier, William Dale, Anna C. Revette, Jane Roberts, Ameya Sanyal, Neha Perumal, Eric C. Blackstone, Hajime Uno, Mary I. Whitehead, Lewis Mustian, Tammy T. Hshieh, Jane A. Driver, Gregory A. Abel

**Affiliations:** 1 Veterans Affairs Boston Healthcare System, New England GRECC, Boston, USA; 2 Brigham and Women’s Hospital, Boston, MA USA; 3 Dana-Farber Cancer Institute, Boston, MA USA; 4 City of Hope National Medical Center, Duarte, CA USA; 5 SCOREboard Stakeholder Advisory Group, Cancer and Aging Research Group, City of Hope, Duarte, CA USA; 6 Center for Bioethics, Harvard Medical School, Boston, MA USA

**Keywords:** Ethics, Oncology, Overtreatment, Undertreatment, Geriatric oncology

## Abstract

**Background:**

Over-/undertreatment are pervasive in older adults with cancer, and challenges arise in applying the principles of bioethics: beneficence, nonmaleficence, justice, and patient autonomy. The objective of this study was to determine whether these ethical principles relate to over-/undertreatment for older adults, and how tensions among the principles may contribute.

**Methods:**

We conducted a modified Delphi study with 13 experts in biomedical ethics for iterative rounds of data collection. In the first round, we presented via electronic questionnaire our previously published definitions of over-/undertreatment in older adults with cancer. We then asked which ethical principles related to each definition, followed by how over-/undertreatment might arise from conflicts among different ethical principles. Consensus for each question was defined as ≥ 75% of experts answering “agree” or “strongly agree”. The second round consisted of a virtual discussion with nine of the panel experts led by a qualitative researcher to summarize round one results and review questions that did not reach consensus, followed by a second questionnaire including those questions.

**Findings:**

Experts reached consensus that beneficence, non-maleficence, and autonomy were related to over-/undertreatment in older adults with cancer. Consensus was reached (92%) that overtreatment can occur when oncologists overemphasize beneficence valuing the potential benefit of cancer treatments, while underemphasizing non-maleficence with respect to treatment toxicities. Consensus was also reached (85%) that undertreatment reflects a lack of justice in equitable consideration of cancer treatments that could provide similar net benefits in older adults compared to younger adults. Lastly, consensus was reached that, in most cases, it is unethical to make a treatment recommendation without (1) formal assessment of patient frailty (e.g., via a geriatric assessment) or (2) the opportunity for the patient to share their values, goals, and preferences.

**Interpretation:**

Our findings elucidate the ethical principles underpinning over- and undertreatment in older adults with cancer.

**Supplementary Information:**

The online version contains supplementary material available at 10.1186/s12910-025-01255-9.

## Introduction

The terms “overtreatment” and “undertreatment” are often used to describe inappropriate management of older adults (age 65 years and older) with cancer. The terms imply that a prescribed treatment strategy is inadequate or excessive compared to some standard criteria defining what *ought *to be prescribed in a patient. However, our prior scoping review found that no standard criteria exist, with treatment guidelines overemphasizing cancer-specific factors while underemphasizing patients’ health status and preferences [1]. We proposed new definitions that account for both cancer-specific factors *and* aging-related health factors important in the care of older patients, as well as patient goals and preferences regarding which benefits and harms are important to them (Table 1) [1, 2]. Although a first step, these definitions require further validation before they can be used as standard criteria to which clinicians, patients, and caregivers can refer.

**Table 1:** Proposed definitions of over-/undertreatment in older adults with cancer

Overtreatment	Treatment of a cancer in an older patient that would not likely lead to symptoms in his/her remaining lifetime OR Intensive treatment of a cancer in a vulnerable^a^ older patient in whom there would be a greater net benefit^a^ from less intensive therapy
Undertreatment	Use of less intensive^b^ cancer treatment in a fit^a^ older adult who would otherwise derive a greater net benefit^c^ from more intensive cancer treatment AND/OR Not providing non-oncologic interventions to improve deficits in geriatric domains^a^ regardless of what cancer therapy is chosen

^a^Fitness/vulnerability as determined by geriatric assessment recommended by the American Society of Clinical Oncology’s Guideline for Geriatric Oncology

^b^Some reduction in a recommended/standard treatment regimen normally used in younger, fit patients

^c^Benefits as jointly defined by the physician and patient outweigh the similarly defined harms resulting from the cancer treatment

However currently understood, over-/undertreatment are thought to be pervasive in older adults with cancer, despite oncologists prescribing with best intentions. Since evidence is limited regarding the benefits and harms of novel therapies in older patients, oncologists and their patients often make treatment choices informed with efficacy data from clinical trials that included only younger and/or healthier patients. What *ought *to be prescribed for older adults in practice—especially those who are frail with multiple comorbidities—is thus not only an empirical question, but also a bioethical one that calls for weighing principles that physicians are taught during medical training: beneficence (maximizing benefit for patients), nonmaleficence (minimizing harms), justice (equitable treatment allocation), and respect for autonomy (respecting patient preferences) [3]. The objective of the current study was to determine whether the principles of bioethics are appropriately reflected in the definitions of over- and undertreatment in older adults with cancer. We further aimed to elucidate how tensions among ethical principles may contribute to over-/undertreatment.

## Methods

### Study design

We designed a modified Delphi study to conduct with experts in biomedical ethics [4]. We defined an expert in biomedical ethics as a professional with dedicated knowledge and skills in bioethics, as indicated by prior bioethics training and/or experience. Purposive sampling prioritized variation in discipline and geographic representation across North American Institutions, with the goal of 10-15 panel experts to ensure reliable consensus [5–9]. We used REDCap, a secure web-based application, to administer questionnaires to participants and collect and analyze responses [10]. We adhered to Recommendations for Conducting and Reporting Delphi Studies (CREDES) [11].

Two older adult cancer survivors from the Cancer and Aging Research Group’s Stakeholders for Care in Oncology and Research for our Elders Board (SCOREboard) contributed to the design and wording of our questionnaire for content validity [12]. Consensus for each question was a priori defined as ≥ 75% of experts answering “agree” or “strongly agree” [13]. Questions that did not reach consensus on the first round were reviewed in a videoconference discussion with all members of the panel and then presented again in a second questionnaire.

### Round 1: electronic questionnaire

Round 1 (Supplemental Figure 1) presented to panelists our definitions of over- and undertreatment in older adults with cancer (Table 1) [1]. We asked panelists to indicate their level of agreement or disagreement regarding statements of whether each of the four bioethical principles was reflected in our definition of overtreatment, and separate statements asking the same for undertreatment [3]. Panelists were asked whether they agreed, disagreed, or were undecided. We then asked panelists to indicate their level of agreement regarding a series of statements depicting how over-/undertreatment might arise from tensions among ethical principles and from challenges in oncologists adhering to their specialty’s professional ethics. Panelists were asked whether they strongly agreed, agreed, disagreed, or strongly disagreed with each statement. A free response item was included at the end. Panelists were blinded to each other’s responses.

### Round 2: virtual discussion session and questionnaire

Answers to Round 1 questions were aggregated to compute percentages, identifying questions that did and did not reach consensus. These questions were asked again in Round 2. The study team reviewed answers in the free response item in Round 1 for any additional aspects regarding the ethics of over-/undertreatment not addressed by the questionnaire, and for any reasons listed by panelists why certain questions did not reach consensus. Findings were used to guide the virtual discussion among panelists; this meeting aimed to resolve conflicts among the questions that did not achieve consensus. The face-to-face meeting (one-hour) was conducted using Zoom secure videoconference software, and professionally transcribed for qualitative analysis. The four ethical principles served as the overarching framework for the analysis, and the qualitative analysis focused on understanding, contextualizing, and summarizing areas of disagreement from Round 1. De-identified data and findings were further reviewed with the two patient advocates. All findings and interpretations were synthesized to inform modifications to the Round 2 questionnaire (Supplemental Figure 2), which was distributed again electronically to all panelists.

## Results

**Fig. 1:**
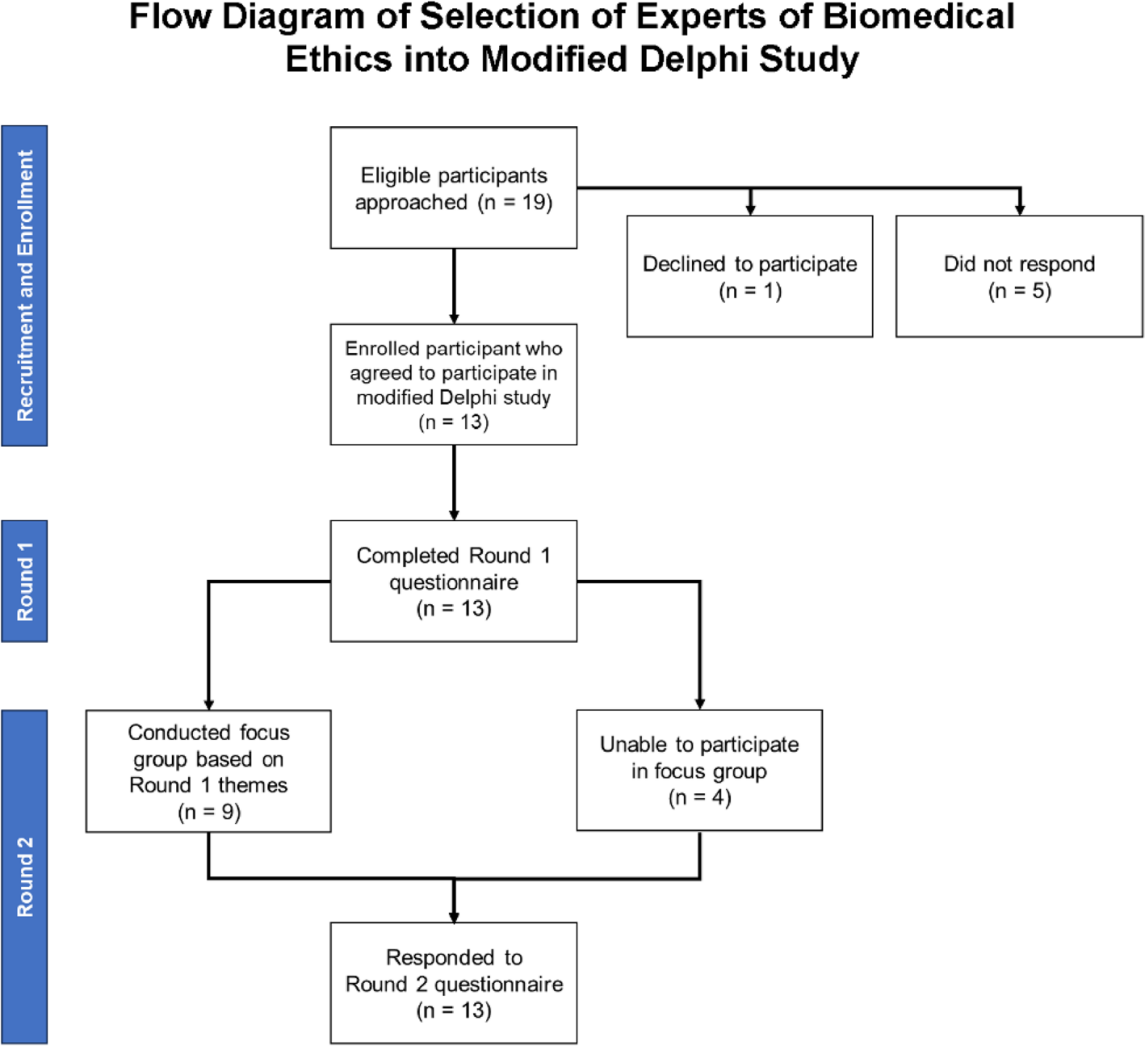
Flow diagram of selection of experts of biomedical ethics into modified Delphi study

After contacting 19 eligible individuals, we convened a panel of 13 experts in biomedical ethics from the U.S. and Canada (five men, eight women, four MDs, four PhDs, two MD/MAs, one MD/PhD, one JD/MDiv, and one DNP; Fig. 1 and Table 2). Four represented oncology specialties, one geriatrics, four internal medicine or medicine subspecialty, one neurology, one in Women’s health, and one in a primary bioethics division.

**Table 2:** Characteristics of experts in biomedical ethics who participated on modified Delphi panel

Institutional Rank	Degree	Region^a^	Medical Discipline	Ethics Affiliation
Lecturer	DNP, RN	Northeast	Pulmonary and Critical Care	Ethics Researcher
Assistant Professor	MD, MSHP	Northeast	Pulmonary and Critical Care	Ethics Committee Member
Assistant Professor	MD, MA	West	Oncology	Ethics Researcher
Assistant Professor	MA, PhD	Canada	Oncology	Ethics Researcher
Associate Professor	JD, MDiv, MA	Mid-Atlantic	Law and Ethics	Ethics Fellow
Associate Professor	PhD, RN, HEC-C	Northeast	Pulmonary and Critical Care	Ethics Committee Member
Associate Professor	MD, MS, FAAN, FAES	Northeast	Neurology	Ethics Researcher
Professor	MD, MSc, MA	Mid-Atlantic	Oncology	Ethics Researcher
Professor	MD	West	Internal Medicine, Geriatrics	Ethics Researcher
Professor	MD, PhD	West	Internal Medicine	Ethics Researcher
Professor	PhD	West	Women's Health	Ethics Center Director, Bioethics Professor
Professor	MD, MS	West	Oncology	Ethics Committee Co-Chair
Professor	PhD	Canada	Bioethics	Ethics Researcher, Ethics Center Director

^a^Region refers to U.S. unless stated as Canada

### Round 1: electronic questionnaire

Table 3 presents the findings of Round 1: ten questions reached consensus, and six did not. Panelists agreed that the principles of beneficence (100%) and non-maleficence (100%) were reflected in our definition of overtreatment, and the principle of beneficence (92%) was reflected in our definition of undertreatment. Ninety-two percent of panel members agreed that overtreatment can occur when oncologists overemphasize beneficence that values potential benefits of cancer treatments, while underemphasizing non-maleficence with respect to treatment adverse effects. Seventy-seven percent felt that overtreatment can also occur when oncologists prioritize patient autonomy (preference to be treated) over non-maleficence (oncologists' concerns that treatment harms outweigh benefits). Eighty-four percent felt that undertreatment can occur due to a lack of justice in equitable consideration of cancer treatments that could provide similar benefits in older adults as they would in younger adults. Moreover, 77% felt that undertreatment can occur when oncologists underemphasize patient autonomy, failing to consider patient preferences regarding which benefits to pursue and which risks to take.

**Table 3:**
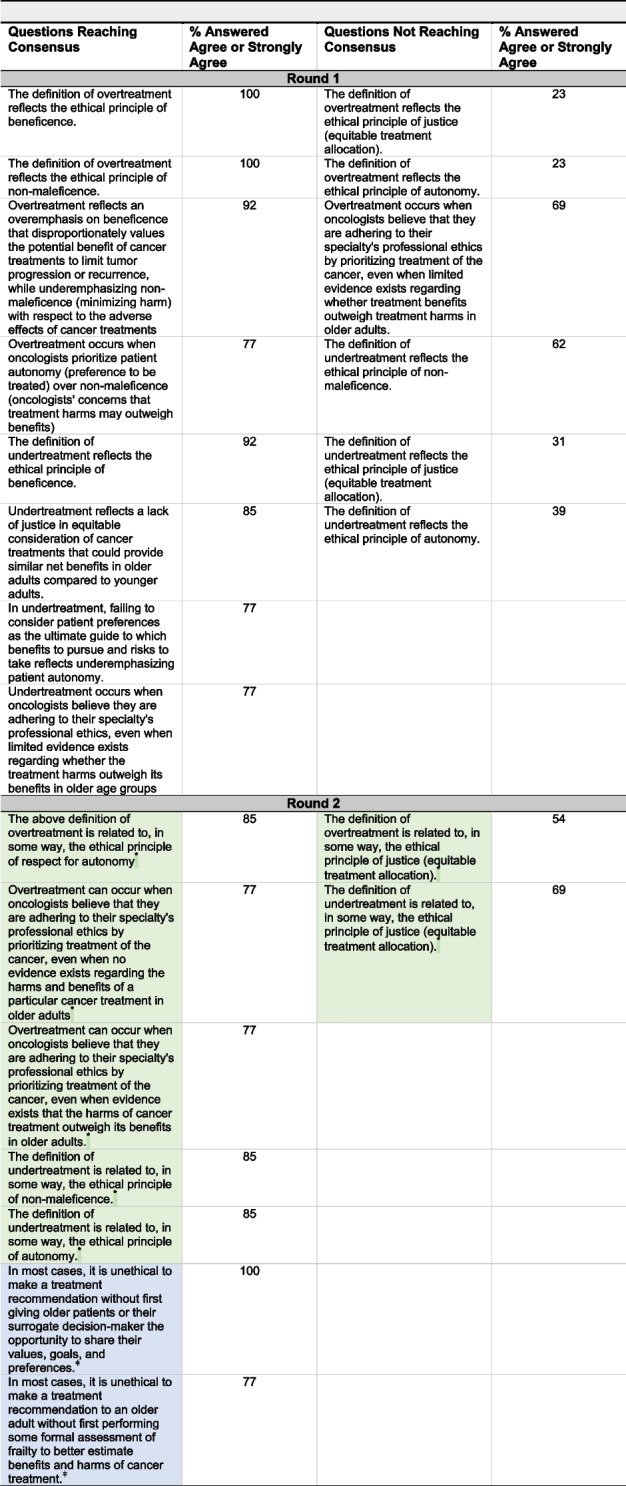
Questions reaching consensus on rounds one and two of Delphi questionnaire. Round 2 questions shaded in green or indicated by "*" were revised versions of Round 1 questions based on findings of the virtual discussion Round 2 questions shaded in blue and indicated by "ǂ" were new questions added based on findings from Round 1 questionnaire and the discussion session

### Round 2: virtual discussion session

Nine of the 13 panelists attended the one-hour virtual synchronous discussion. An overview of the study was given at the beginning, as was a review of the definitions of over- and undertreatment. The four bioethics principles were also reviewed, as were the questions from the first round that met consensus. The remaining time was spent discussing questions that did not reach consensus and reviewing two new questions to include in Round 2 questionnaire.

#### Free response comments

First, some panelists expressed concern that the definitions of over- or undertreatment did not reflect *adherence* to an ethical principle, but rather a *violation* of it. The research team clarified that the goal was to determine if the ethical principles were related in some way to our definitions of over- or undertreatment; this relationship could be either *adherence to* or *violation of* an ethical principle. Second, some panelists expressed confusion regarding whether the ethics concerning over-/undertreatment should be applied to treatment decisions on the individual patient-level versus on a health system-wide or population level. The research team clarified that, for the current study, panelists were being asked to consider ethical implications concerning decisions that involve individual older adults.

#### Justice

Panelists gave a mixed response when asked whether our definitions of over- and undertreatment related to the principle of justice. Overall, many felt that justice was too broad of a construct to be encapsulated by our definitions. For example, one panelist mentioned that a more accurate interpretation of justice would be that *no patient of any age* should be over- or undertreated—a goal not restricted to older patients. Moreover, overtreating older adults may draw limited resources—such as cancer treatments and payor reimbursements—away from other patients in the healthcare system. This potential misallocation of resources due to overtreatment could represent a lack of justice on a population level. Finally, our definitions did not reference social determinants of health that are often considered violations of justice, such as a lack of access to cancer centers for older patients with mobility limitations or challenges with travel for those living in rural areas. Nonetheless, there was wide agreement among panelists that a lack of justice is associated with undertreatment when general treatment guidelines or rules are applied without individualization.

#### Beneficence and nonmaleficence

Whereas not prescribing beneficial treatments based solely on age represented a lack of justice, prescribing treatments to older adults based solely on guidelines was agreed to represent an overemphasis on potential treatment benefits and an underemphasis on potential treatment harms—drawing from the principles of beneficence and nonmaleficence, respectively. There was general agreement that nonmaleficence was central to the definition of overtreatment, interwoven into its criteria and paramount when considering novel cancer treatments in frail older adults in whom treatment toxicity may outweigh treatment benefits.

#### Autonomy

One panelist called for the principle of autonomy to be qualified as “respect for autonomy,” a sentiment that was generally agreed upon by the other panelists. Another panelist felt that respect for autonomy should be elevated as the most important principle that governed all others. This prioritization of autonomy was then debated, and there were some who wished to place bounds on autonomy. A panelist expressed that autonomy should only be considered within a range of “medically appropriate” treatment options. These options should first be determined by the patient’s oncology team, which has the professional expertise and knowledge of the latest available evidence regarding the benefits and harms of cancer treatments. On the other hand, if an older patient chooses to decline a treatment even when the oncologist believes it is of benefit, panelists agreed that this choice must be respected under the principle of autonomy.

Two new questions—developed based on findings from Round 1—received broad agreement from the panel, with minor wording changes: “In most cases, it is unethical to make a treatment recommendation without first giving older patients or their surrogate decision-maker the opportunity to share their values, goals, and preferences,” and, “In most cases, it is unethical to make a treatment recommendation to an older adult without first performing some formal assessment of frailty to better estimate benefits and harms of cancer treatment.” Table 4 lists select quotes from the Round 1 free responses and the Round 2 virtual discussion.

**Table 4:** Select quotes from answers to the free response item on the questionnaire and the virtual discussion on the ethical principles involved in over- and undertreatment

*On justice:* “For example, a rule that anyone over 70 gets reduced-dose chemotherapy without looking at their individual comorbidities and risk factors is in conflict with the principle of justice, and that you may be violating the principle of justice by undertreating patients, just based on a number, rather than looking at the entire picture. And, likewise, you shouldn’t just treat all young patients with intensive chemotherapy, if it violates their principles.”
*On justice:* “How responsive the definitions are to—e.g., social justice—will be in how the definitions are applied and used, how much weight and what criteria are embedded within the geriatric assessment, and how that assessment is linked to the definitions. There are naturally bound to be implicit, or'behind-the-scenes'priority judgments and value judgments that can't easily be controlled via tidy definitions. One approach is to acknowledge this and try to set up some ethical guardrails in an accompanying contextual/applied guidance to advise those relying on the definitions.”
*On nonmaleficence and beneficence:* “I find it very confusing a separation of nonmaleficence from beneficence. Maybe other people don’t, but I find this always very puzzling.”
*On autonomy:* “Because it really seems like under your definitions the place where the patient’s individual autonomy fits in is in the definition of benefit. And once benefit is defined, then it seems like the determination of whether a patient is being overtreated or undertreated is somewhat out of their hands. It seems to me like there might be situations in which a patient might acknowledge that they could benefit more through treatment and yet not want treatment. And I’m not sure that I would consider that to be undertreatment, if that makes sense.”
*On autonomy:* “It seems important to include patient request that may differ from expert opinion - for example, the clinician who opts to provide immunotherapy per patient/family request NOT because they think the pt will benefit but because they think it"can't hurt"and will appease the patient/family. This is a common source of over-treatment in my experience.”
*On overtreatment when oncologists believe they are adhering to their specialty’s professional ethics despite limited evidence on benefits and harms:* “Maybe if [the question] said, even when there’s no evidence, that would be easier for me to agree with. But if there’s limited evidence, what does that mean, right? So extrapolating, which I do a lot, is that limited evidence or is that using what we know?” “But when there’s evidence that the harm outweighs the benefit, then that’s where overtreatment occurs, not when there’s necessarily limited evidence but when there’s clear evidence that the harm outweighs the benefits.”
*On patient preferences:* “Over- and undertreatment can also result from a failure to elicit patient preferences regarding which benefits to pursue/harms to avoid.”
*On formal frailty assessment:* “I also wonder whether it is helpful to recognize that failing to perform a GA [geriatric assessment] when indicated could result in either over- or undertreatment.”

### Round 2: questionnaire

In addition to adding the two new questions on patient preferences and frailty, we made changes to the questions that did not reach consensus based on the virtual discussion. In the questions asking whether each ethical principle was reflected in our definitions, we changed “reflects” to “related to.” We further changed the principle of “autonomy” to “respect for autonomy.” For the question, “Overtreatment occurs when oncologists believe that they are adhering to their specialty's professional ethics by prioritizing treatment of the cancer, even when limited evidence exists regarding whether treatment benefits outweigh treatment harms in older adults,” we exchanged with two new questions ending with “... even when *no* evidence exists regarding the harms and benefits of a particular cancer treatment in older adults,” and, “... even when evidence exists that the harms of cancer treatment outweigh its benefits in older adults.”

All thirteen panelists participated in the Round 2 questionnaire that presented the revised questions. All questions achieved consensus (Table 3), except for two questions asking whether our definitions of over-/undertreatment related in some way to the principle of justice.

## Discussion

This modified Delphi study convened a panel of experts in biomedical ethics and reached consensus that the principles of beneficence, non-maleficence, and autonomy are related to our previously proposed definitions of over- and undertreatment in older adults with cancer. The panel also reached consensus that, in most cases, it is unethical to make a treatment recommendation without (1) formal assessment of patient frailty (e.g., via a geriatric assessment) and (2) the opportunity for a patient to share their values, goals, and/or preferences. The panel did not reach consensus regarding the relationship between justice and over-/undertreatment; however, the panel concluded that justice applies to undertreatment when an oncologist withholds potentially beneficial cancer treatment in an older patient based on their age alone.

Tensions among bioethical principles can lead to over- and undertreatment in older adults with cancer (Table 5). With near unanimous agreement on the first round, the panel reached consensus that valuing the principle of beneficence over nonmaleficence can lead to overtreatment. This overemphasis on beneficence stems from biomedical reductionism, focusing more on the cancer-centric benefits of treatment rather than the impact of a treatment on the whole patient [14]. The panel agreed that the criteria of our definition of overtreatment appropriately elevated the overlooked principle of nonmaleficence. The concept of “minimizing harm” in older patients—especially those who are frail—must be weighed equally alongside “maximizing benefit” in terms of cancer control [2].

**Table 5:** Tensions noted in ethical principles leading to over-/undertreatment in older adults with cancer

Tension in ethical principles	Effect	Example	Solutions to resolve tension
Beneficence > Nonmaleficence	Overtreatment	Oncologist prescribes intensive treatment in an older patient based on evidence suggesting its ability to improve progression free survival, neglecting the reduced physiologic reserve of the older patient that puts them at higher risk of toxicity and lower overall survival	Perform frailty assessment (using geriatric assessment) to better gauge whether risks of intensive treatment outweigh tumor-specific benefits. If patient is frail, consider less intensive dosing, frequency, or alternative drugs that offer similar benefit at lower risk of harm^,^
Autonomy > Nonmaleficence	Overtreatment	Oncologist prescribes treatment in older patient to appease their (or their family’s) desire to receive treatment, despite the oncologist’s concern that treatment harms outweigh benefits	Share with patient/family the findings of frailty assessment along with cancer-specific information. Suggest less intensive alternative treatment option that is safer while still providing benefit in line with the patient’s stated goals
Beneficence > Autonomy	Overtreatment	Oncologist prescribes intensive cancer treatment with high risk of side effects in an older patient who wishes to avoid aggressive therapies and values quality of life over life prolongation	Obtain patient goals and preferences and recommend less intensive option that provides some degree of cancer control while minimizing threats to function and quality of life
Nonmaleficence > Autonomy	Undertreatment	Oncologist prescribes less intensive treatment option in an older patient who prioritizes cancer control and prevention of progression or recurrence	Obtain patient goals and preferences, and offer similar guideline-recommend treatment that is used in younger patients
Nonmaleficence > Justice	Undertreatment	Oncologist withholds cancer treatment in an older patient simply because of their age	Perform frailty assessment to judge benefits and harms based on patient physiology, not numeric age

The panel reached consensus that overtreatment can also occur when oncologists prescribe cancer treatment out of a belief they are adhering to their specialty's professional ethics, even when there is no evidence regarding treatment effects in older patients, or when existing evidence suggests that harms outweigh benefits. Providing a less intensive treatment option—including different drugs, reduced dosing, reduced frequency, or no treatment altogether—may be seen as failing to uphold a professional duty to provide patients treatment options that maximize cancer control [15, 16]. Oncologists may therefore view that cancer recurrence or progression in an older patient prescribed a less intensive treatment option poses a greater risk than the increased toxicity resulting from a more intensive treatment option. This calculus in decision-making has even been called the “Oncologist’s Wager”, an allusion to the historical “Pascal’s Wager” that concluded that the severity of the potential negative consequences of not pursuing an action (in the case of oncology, cancer spreading or recurring after a recommendation for less intensive or no treatment) were always greater than the potential negative consequences of pursuing an action (in oncology, toxicity after a recommendation to treat cancer) [17]. One implication of our findings is that by reasserting the principle of nonmaleficence alongside the principle of beneficence, oncologists will better adhere to their specialty’s professional ethics when they acknowledge that a given treatment’s harms may outweigh its benefits in frail older patients. In these scenarios, prescribing a less intensive treatment is not a form of undertreatment, but rather an ethically appropriate recommendation that prevents overtreatment.

The panel also reached consensus that tensions between autonomy and other principles can lead to both overtreatment and undertreatment. When nonmaleficence overrides autonomy, undertreatment can occur if an oncologist presumes that an older adult would not accept the risks of cancer treatment without first clarifying patient goals and preferences. When beneficence overrides autonomy, overtreatment can occur if an oncologist presumes the potential benefits of cancer treatment are desirable and prescribes treatment without clarifying goals and preferences. As reinforced by the panel, an older adult has the right to decline a treatment even when the oncologist believes it is of benefit. In this scenario, considering a less intensive option, or no cancer treatment at all, is not a form of undertreatment, but rather an ethically appropriate recommendation that respects patient autonomy.

Conversely, when autonomy overrides nonmaleficence, overtreatment can occur if an oncologist appeases the desire of an older patient (or patient’s family) to receive an intensive cancer treatment, despite the oncologist’s concern that the treatment’s harms likely outweigh its benefits. The overemphasis on preserving patient autonomy in modern medicine has even been referred to as the “tyranny of autonomy”; respecting patient preferences does not imply elevating their superiority in all cases above oncologists’ expertise [18]. Even if an older patient (or their family) requests an intensive treatment option, an oncologist’s recommendation for a less intensive alternative respects both autonomy and nonmaleficence if the oncologist believes the alternative to be a safer option that provides a benefit better aligned with the patient’s stated goals.

Regarding justice, the panel concluded that withholding a guideline-recommended therapy based on age alone exemplifies a lack of justice. Our definitions of over-/undertreatment advocate for the use of fitness/frailty when evaluating benefits and harms of treatments in older adults, avoiding arbitrary cutoffs based solely on chronologic age [19]. The panel’s inability to reach consensus on relating our definitions to the principle of justice as a whole stems from justice’s inherent call to consider other patients in the healthcare system when making individual-level treatment decisions (Supplemental Figure 3). This aspect of justice stands in contrast to autonomy, beneficence, and nonmaleficence, which are more readily applied to decisions in individual patients. Nonetheless, the virtual discussion session provided insight into how overtreatment of older adults unlikely to benefit from an intensive regimen may lead to undertreatment of other patients in the healthcare system (Supplemental Figure 3B). Moreover, if an older adult who could benefit from cancer treatment cannot access it due to mobility or functional limitations, geographic distance, or unreliable transportation, this lack of access reflects system-level inequalities that contribute to undertreatment (Supplemental Figure 3A) [20]. The same applies to an older adult unable to access a geriatric assessment and interventions that in turn leads to suboptimal cancer treatment [21].

Leading cancer organizations such as the American Society of Clinical Oncology (ASCO) now recommend formal assessment of frailty, by way of a geriatric assessment in older adults undergoing systemic cancer treatment [22, 23]. Our panel concluded that, in most cases, prescribing cancer treatment without frailty assessment raises ethical concerns. Frailty assessment shifts a cancer-centric view of treatment effects to a more holistic, patient-centered view, one that considers whether treatment benefits and harms differ in frail older adults compared to the fitter and/or younger patients who are overrepresented in clinical trials [24]. Evaluation of benefits and harms in light of an older adult’s physiology mitigates the risk of making treatment decisions based on age alone, which violates justice. This shift to a more patient-centered evaluation of benefits and harms also resolves the tension between beneficence and nonmaleficence, since it encourages weighing the tumor-specific benefits of treatment against the risk of treatment toxicity [25, 26]. At the same time, simply labeling a patient as “frail” (e.g., from gestalt and not after formal geriatric assessment) and withholding guideline-based treatments solely on that basis can reflect undertreatment—just as it can when decisions are based only on chronological age. This is especially problematic when a treatable cancer is the main cause of a patient’s frailty, or when addressing other frailty factors could improve tolerance to beneficial cancer therapies [27].

In this vein, a more patient-centered evaluation via formal frailty assessment reveals other health deficits (e.g., comorbidities, cognitive impairment, or functional limitations) that often present concurrently with cancer in older patients. Our definition of undertreatment includes the failure to recognize and optimize these nononcologic health deficits, since their optimization can mitigate toxicity risk and promote adherence to cancer treatment [28]. Recent randomized controlled trials conclude that oncology care that is guided by frailty assessment (compared to standard oncology care) informs anti-cancer treatment modifications and/or supportive care interventions that mitigate overtreatment of an older adult’s cancer and undertreatment of their nononcologic conditions [29–31]. Evidence from trials is more limited regarding the effectiveness of frailty assessment in improving long-term survival, as the survival outcomes are assessed at 6-month and 12-month endpoints in the large RCTs. Also, caution should be exercised against reflexive treatment de-escalation simply because a patient has one or more health deficits found on geriatric assessment. Geriatric assessment is meant to augment and improve clinical judgment, not supersede it. Just as withholding guideline-recommended treatments based only on labeling a patient as “frail” can reflect undertreatment, withholding guideline treatments based only on the presence of functional or cognitive deficits may also reflect undertreatment, especially if the patient prioritizes life prolongation above other outcomes.

Moreover, identifying and addressing deficits such as hearing and/or cognitive impairment early in the treatment decision process can also better ensure that oncology teams deliver fair and informed communication. Evaluating deficits includes considering the ability of the older patient to receive information on treatment decisions, to weigh risks and benefits of different options, and to ascertain his/her overall capacity to accept or reject a treatment recommendation. Indeed, formal frailty assessment has been shown to (1) raise awareness of aging-related concerns among oncologists and (2) improve their communication with older patients and caregivers [32, 33]. Since the way in which an oncologist presents different treatment options plays a determining role in the final treatment decision, a formal frailty assessment of health domains essential to older adult well-being expands a cancer-centric discussion to a more patient-centered discussion.

The panel also reached unanimous consensus that, in most cases, it is unethical to prescribe cancer treatment without first offering older patients the opportunity to share their goals and preferences. Whereas some older patients prioritize cancer control just as much as younger patients, other older adults prioritize maintaining independence and avoiding time spent in the hospital [34]. In the latter case, aggressive treatments may present more harm than benefit if their side effects jeopardize function and quality of life—even if the treatments lengthen life [35]. Evidence suggests that there is often inadequate communication regarding goals of care between patients, families, and oncologists, undermining autonomy and potentially contributing to over-/undertreatment [36–38]. Multiple interventions have been tested to improve goals of care communication in oncology, such as decision aids and communication training for oncologists [39, 40]. Our findings underscore the importance of these efforts, particularly in older patients for whom benefits of intensive therapy have not been established. Our proposed criteria of over-/undertreatment require defining the “benefits” older adults wish to pursue and the “harms” they wish to avoid. Obtaining these goals and preferences reasserts respect for patient autonomy, resolving tensions between this principle and others.

There are limitations to this study. First, we did not explore other ethical frameworks that may apply to our definitions of over-/undertreatment. We focused on the Beauchamp and Childress principles that are the most widely taught and recognized frameworks among the medical community. Although the tensions that can arise among these ethical principles are often cited as a limitation for their use in guiding treatment decisions, our work reveals that identifying these tensions can better explain how over- and undertreatment occurs. Second, our Delphi panel consisted of experts in biomedical ethics from North American institutions. Lack of representation outside of North America may influence the generalizability of our findings, and different countries and cultures may vary the application of ethical principles to decision-making in older patients. Finally, the very attempt to apply ethical standards to better define over- or undertreatment in older adults may appear problematic, since the benefits and harms of novel treatments in older adults are often uncertain. However, treatment decisions must still be made despite this uncertainty, and the lack of evidence to guide such decisions necessitates adherence to bioethics.

In conclusion, this work establishes the ethical principles underlying over-/undertreatment in older adults with cancer. The identification of relevant tensions among these principles is a vital step towards reducing over-/undertreatment, ensuring older patients receive appropriate care when facing limited evidence. Our findings suggest an ethical imperative to scale formal assessment of frailty via the geriatric assessment for more widespread use in oncology practice, as well as evaluation of patient goals and preferences.

## Supplementary Information

Supplementary Material 1. Supplemental Figure 1: Delphi Round 1 Questionnaire.

Supplementary Material 2. Supplemental Figure 2: Delphi Round 2 Questionnaire.

Supplementary Material 3. Supplemental Figure 3: Individual and Population-Level Aspects of Justice.

## Data Availability

Data requests will be considered on reasonable request consistent with the protocols of the Dana-Farber/Harvard Cancer Center Institutional Review Board.

## Abbreviations

CREDES: Recommendations for Conducting and Reporting Delphi Studies
SCOREboard: Stakeholders for Care in Oncology and Research for our Elders Board
GA: Geriatric assessment
ASCO: American Society of Clinical Oncology
