# Supplementary material for: Ethics of overtreatment and undertreatment in older adults with cancer
Source: BMC Med Ethics. 2025 Jul 24;26:105. doi: 10.1186/s12910-025-01255-9 (PMC12291383; doi:10.1186/s12910-025-01255-9)
Supplement: Supplementary file 1 — Supplementary Material 1. Supplemental Figure 1: Delphi Round 1 Questionnaire. [file 12910_2025_1255_MOESM1_ESM.pdf]

Round 1 Questionnaire: Ethics of Over- and Undertreatment

This goal of this study is to elucidate the ethical foundations of overtreatment and undertreatment of older patients with cancer through a modified Delphi panel. Your expertise in bioethics is valuable to establish expert consensus regarding our definitions of over-/undertreatment in older adults with cancer and our decision-making framework that aims to minimize both.

- This first-round questionnaire will take approximately 10 minutes.
- This questionnaire is confidential; identifiable information will be removed and responses aggregated.
- You may quit the questionnaire at any time and for any reason.
- If you come across a question you would rather not answer, it is okay to skip it and move on to the next question.
- There are no direct benefits to participating. A $100 gift card will be provided as a thank you for your participation in the study once it concludes if you participate in the focus group.

We anticipate that the findings of this study will inform standard definitions of over- and undertreatment that are well-grounded in medical ethics, and consider quality of life and functional outcomes for patients with cancer. There are no additional anticipated risks from participation except possible discomfort with the topic. If you have any questions or concerns about the research, you can contact the study's co-principal investigator, Dr. Clark DuMontier at 920-740-2790 or cdumontier@bwh.harvard.edu.

This section will focus on the ethical principles of our definition of overtreatment.

For the following statements regarding our definition of overtreatment, please mark whether you agree, disagree, or are undecided about the bioethical principle being reflected.

Definition of Overtreatment:
"Treatment of a cancer, that would not likely lead to symptoms in his/her remaining lifetime, in an older patient OR intensive treatment of a cancer in a vulnerable* older patient in whom there would be a greater net benefit** from less intensive therapy."

*Fitness/vulnerability as determined by geriatric assessment recommended by the American Society of Clinical Oncology's Guideline for Geriatric Oncology.

**Benefits as jointly defined by the physician and patient outweigh the similarly defined harms resulting from the cancer treatment. Examples of benefits include prolonging survival and maintaining quality of life. Examples of harm include cancer recurrence or side effects of cancer treatment.

The above definition of overtreatment reflects the ethical principle of beneficence (maximizing benefits).

○ Agree ○ Disagree ○ Undecided

The above definition of overtreatment reflects the ethical principle of non-maleficence (minimizing harms).

○ Agree ○ Disagree ○ Undecided

The above definition of overtreatment reflects the ethical principle of justice (equitable treatment allocation).

○ Agree ○ Disagree ○ Undecided

The above definition of overtreatment reflects the ethical principle of autonomy (respecting patient preferences).

○ Agree ○ Disagree ○ Undecided

Which ethical principle is most relevant to our definition of overtreatment?

◯ Beneficence ◯ Non-maleficence ◯ Justice ◯ Autonomy

Please let us know to what extent you agree with the following statements.

Overtreatment reflects an overemphasis on beneficence that disproportionately values the potential benefit of cancer treatments to limit tumor progression or recurrence, while underemphasizing non-maleficence (minimizing harm) with respect to the adverse effects of cancer treatments.

◯ Strongly agree ◯ Agree ◯ Disagree ◯ Strongly Disagree

Overtreatment occurs when oncologists believe that they are adhering to their specialty's professional ethics by prioritizing treatment of the cancer, even when limited evidence exists regarding whether treatment benefits outweigh treatment harms in older adults.

◯ Strongly agree ◯ Agree ◯ Disagree ◯ Strongly Disagree

Overtreatment occurs when oncologists prioritize patient autonomy (preference to be treated) over non-maleficence (oncologists' concerns that treatment harms may outweigh benefits).

◯ Strongly agree ◯ Agree ◯ Disagree ◯ Strongly Disagree

This section will focus on the ethical principles of our definition of undertreatment.

For the following statements regarding our definition of undertreatment, please mark whether you agree, disagree, or are undecided about the bioethical principle being reflected.

Definition of Undertreatment
"Use of less intensive* cancer treatment in a fit** older adult who would otherwise derive a greater net benefit*** from more intensive cancer treatment AND/OR not providing non-oncologic interventions to improve deficits in geriatric domains** regardless of what cancer therapy is chosen"

*Some reduction in a recommended/standard treatment regimen normally used in younger, fit patients.

**Fitness/vulnerability as determined by geriatric assessment recommended by the American Society of Clinical Oncology's Guideline for Geriatric Oncology.

***Benefits as jointly defined by the physician and patient outweigh the similarly defined harms resulting from the cancer treatment. Examples of benefits include prolonging survival and maintaining quality of life. Examples of harm include cancer recurrence or side effects of cancer treatment.

The above definition of undertreatment reflects the ethical principle of beneficence (maximizing benefits).

◯ Agree ◯ Disagree ◯ Undecided

The above definition of undertreatment reflects the ethical principle of non-maleficence (minimizing harms).

◯ Agree ◯ Disagree ◯ Undecided

The above definition of undertreatment reflects the ethical principle of justice (equitable treatment allocation).

◯ Agree ◯ Disagree ◯ Undecided

The above definition of undertreatment reflects the ethical principle of autonomy (respecting patient preferences).

○ Agree ○ Disagree ○ Undecided

Which ethical principle is most relevant to our definition of undertreatment?

○ Beneficence ○ Non-maleficence ○ Justice ○ Autonomy

Please let us know to what extent you agree with the following statements.

Undertreatment reflects a lack of justice in equitable consideration of cancer treatments that could provide similar net benefits in older adults as they would in younger adults.

○ Strongly Agree ○ Agree ○ Disagree ○ Strongly Disagree

In undertreatment, failing to consider patient preferences as the ultimate guide to which benefits to pursue and risks to take reflects underemphasizing patient autonomy.

○ Strongly Agree ○ Agree ○ Disagree ○ Strongly Disagree

Undertreatment occurs when oncologists believe that they are adhering to their specialty's professional ethics by limiting toxicity-inducing treatment of cancer in older patients based on their advanced age, even when limited evidence exists regarding whether the treatment harms outweigh its benefits in older age groups.

○ Strongly agree ○ Agree ○ Disagree ○ Strongly Disagree

Are there any other ethical considerations that you feel are important relating to over- and undertreatment in older adults with cancer? Should you wish, you may also use this space to elaborate more on any of your answer choices from before.

Please fill out the following demographic information.

First Name

____

Last Name

____

Preferred Email

____

Institution

____

Please fill in your highest degree, and the area of concentration.

____

To which gender identity do you most identify?

◯ Male
◯ Female
◯ Prefer to self describe

____

Race

☐ American Indian or Alaskan Native
☐ Asian
☐ Black or African American
☐ Native Hawaiian or Other Pacific Islander
☐ White
☐ Prefer not to answer

Are you of Hispanic or Latino origin?

◯ Yes
◯ No

Can you attend a follow-up focus group session hosted at any of these times?

☐ Tuesday, October 3rd, 3 - 4:30PM EST
☐ Tuesday, October 3rd, 4 - 5:30PM EST
☐ None of these options work for me
