# Supplementary material for: Ethics of overtreatment and undertreatment in older adults with cancer
Source: BMC Med Ethics. 2025 Jul 24;26:105. doi: 10.1186/s12910-025-01255-9 (PMC12291383; doi:10.1186/s12910-025-01255-9)
Supplement: Supplementary file 2 — Supplementary Material 2. Supplemental Figure 2: Delphi Round 2 Questionnaire. [file 12910_2025_1255_MOESM2_ESM.pdf]

Round 2 Questionnaire: Ethics of Over- and Undertreatment

As a reminder, the goal of this study is to elucidate ethical foundations of overtreatment and undertreatment of older patients with cancer through a modified Delphi approach.

This second questionnaire contains the items that did not reach consensus in the first round, with revisions to the wording of the questions based on our discussion in the focus group. We also included two new questions at the end that were based on free responses in the first round and findings from our focus group.

• This second-round questionnaire will take approximately 10 minutes.

• This questionnaire is confidential; identifiable information will be removed and responses aggregated.

• You may quit the questionnaire at any time and for any reason.

• If you come across a question you would rather not answer, it is okay to skip it and move on to the next question.

• There are no direct benefits to participating. A $100 gift card will be provided as a thank you for your participation in the study once it concludes.

We appreciate the feedback related to the wording of the definitions of overtreatment and undertreatment themselves, and we will make any revisions to these definitions once the modified Delphi study is complete.

If you have any questions or concerns about the research, you can contact the study's co-principal investigator, Dr. Clark DuMontier at 920-740-2790 or cdumontier@bwh.harvard.edu.

For the following statements regarding our definition of overtreatment, please mark whether you agree, disagree, or are undecided.

Definition of Overtreatment:
"Treatment of a cancer, that would not likely lead to symptoms in his/her remaining lifetime, in an older patient OR intensive treatment of a cancer in a vulnerable* older patient in whom there would be a greater net benefit** from less intensive therapy."

*Fitness/vulnerability as determined by geriatric assessment recommended by the American Society of Clinical Oncology's Guideline for Geriatric Oncology.

**Benefits as jointly defined by the physician and patient outweigh the similarly defined harms resulting from the cancer treatment. Examples of benefits include prolonging survival and maintaining quality of life. Examples of harm include cancer recurrence or side effects of cancer treatment.

The above definition of overtreatment is related to, in some way, the ethical principle of justice (equitable treatment allocation).

◯ Agree ◯ Disagree ◯ Undecided

The above definition of overtreatment is related to, in some way, the ethical principle of respect for autonomy (respecting patient preferences).

◯ Agree ◯ Disagree ◯ Undecided

Please let us know to what extent you agree with the following statements.

Overtreatment can occur when oncologists believe that they are adhering to their specialty's professional ethics by prioritizing treatment of the cancer, even when no evidence exists regarding the harms and benefits of a particular cancer treatment in older adults.

◯ Strongly agree ◯ Agree ◯ Disagree ◯ Strongly Disagree

Overtreatment can occur when oncologists believe that they are adhering to their specialty's professional ethics by prioritizing treatment of the cancer, even when evidence exists that the harms of cancer treatment outweigh its benefits in older adults.

◯ Strongly agree ◯ Agree ◯ Disagree ◯ Strongly Disagree

For the following statements regarding our definition of undertreatment, please mark whether you agree, disagree, or are undecided.

Definition of Undertreatment
"Use of less intensive* cancer treatment in a fit** older adult who would otherwise derive a greater net benefit*** from more intensive cancer treatment AND/OR not providing non-oncologic interventions to improve deficits in geriatric domains** regardless of what cancer therapy is chosen"

*Some reduction in a recommended/standard treatment regimen normally used in younger, fit patients.

**Fitness/vulnerability as determined by geriatric assessment recommended by the American Society of Clinical Oncology's Guideline for Geriatric Oncology.

***Benefits as jointly defined by the physician and patient outweigh the similarly defined harms resulting from the cancer treatment. Examples of benefits include prolonging survival and maintaining quality of life. Examples of harm include cancer recurrence or side effects of cancer treatment.

The above definition of undertreatment is related to, in some way, the ethical principle of non-maleficience (minimizing harms).

◯ Agree ◯ Disagree ◯ Undecided

The above definition of undertreatment is related to, in some way, the ethical principle of justice (equitable treatment allocation).

◯ Agree ◯ Disagree ◯ Undecided

The above definition of undertreatment is related to, in some way, the ethical principle of respect for autonomy (respecting patient preferences).

◯ Agree ◯ Disagree ◯ Undecided

Please let us know to what extent you agree with the following statements.

In most cases, it is unethical to make a treatment recommendation without first giving older patients or their surrogate decision-maker the opportunity to share their values, goals and preferences.

◯ Strongly agree ◯ Agree ◯ Disagree ◯ Strongly Disagree

In most cases, it is unethical to make a treatment recommendation to an older adult without first performing some formal assessment of frailty to better estimate benefits and harms of cancer treatment.

◯ Strongly agree ◯ Agree ◯ Disagree ◯ Strongly Disagree
