# Supplementary material for: Ethics of overtreatment and undertreatment in older adults with cancer
Source: BMC Med Ethics. 2025 Jul 24;26:105. doi: 10.1186/s12910-025-01255-9 (PMC12291383; doi:10.1186/s12910-025-01255-9)
Supplement: Supplementary file 3 — Supplementary Material 3. Supplemental Figure 3: Individual and Population-Level Aspects of Justice. [file 12910_2025_1255_MOESM3_ESM.docx]

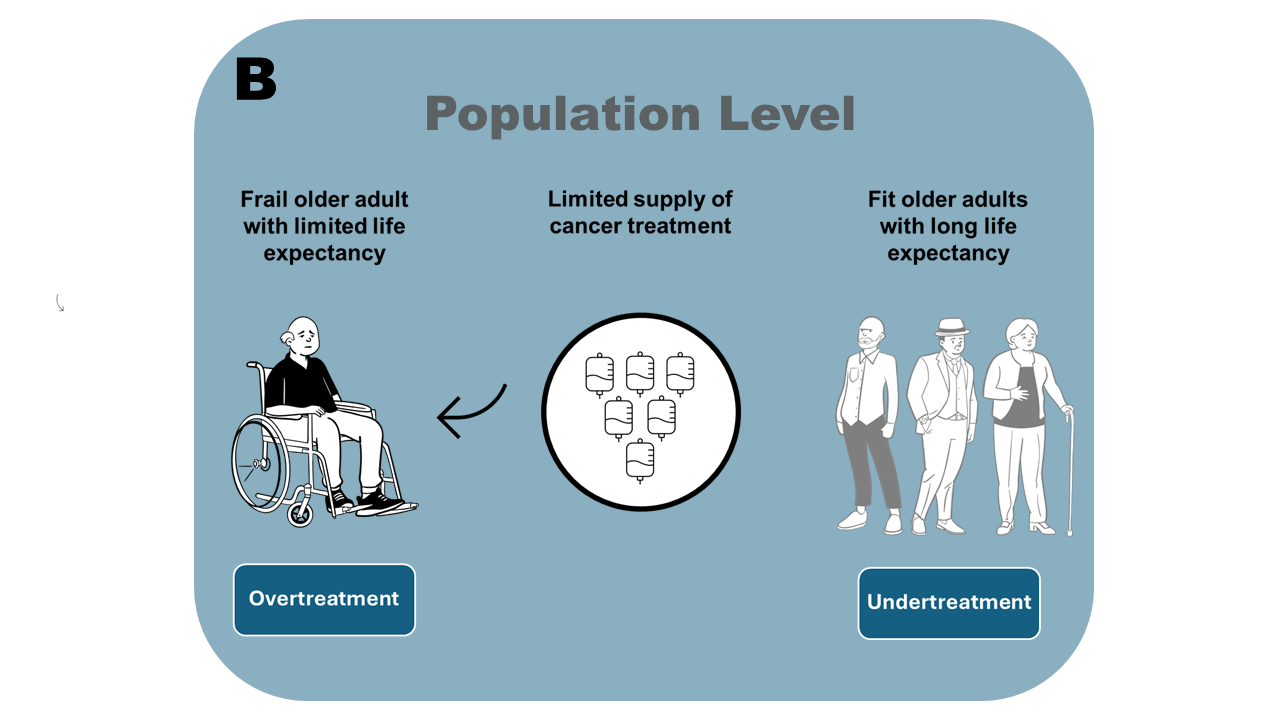

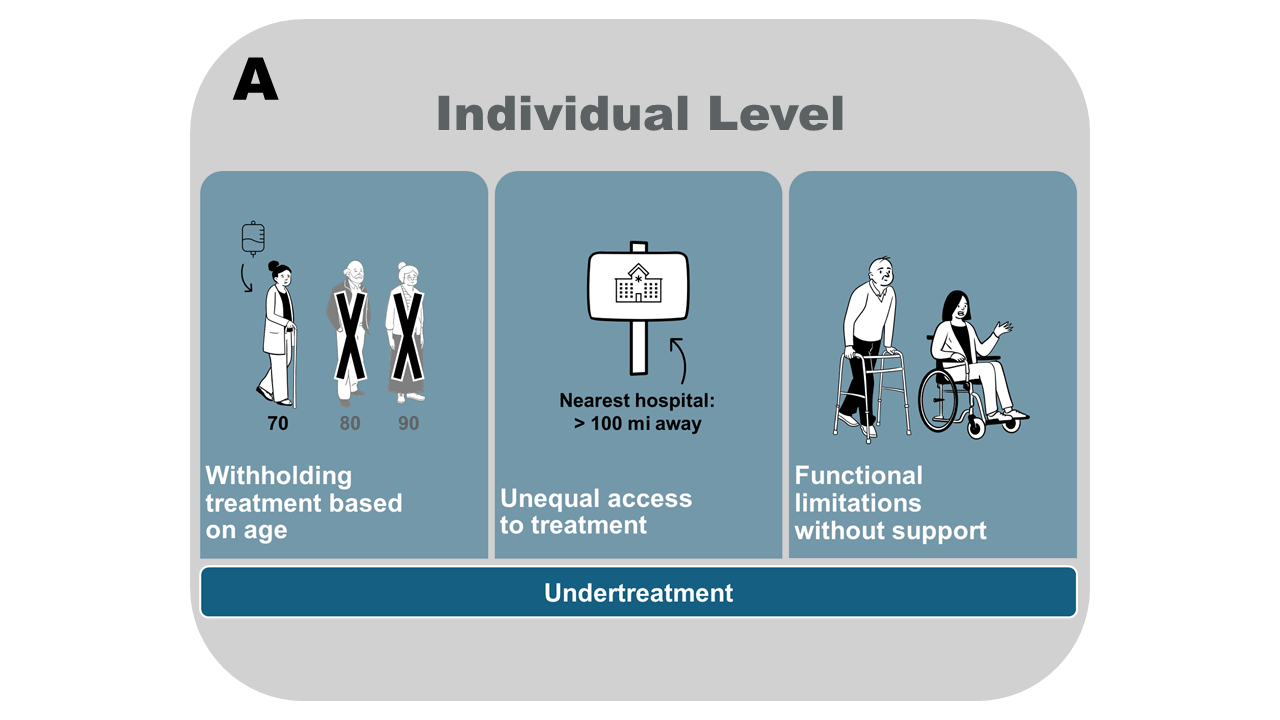
**Supplemental Figure 3**: Aspects of Justice concerning individual (**A**) and population-level (**B**) considerations related to over-/undertreatment of older adults with cancer. **A**: Undertreatment of older adults could result from decisions based on chronologic age alone, lack of access to cancer treatment, and/or functional limitations without adequate support. **B:** Overtreatment of older adults unlikely to benefit from an intensive regimen may lead to undertreatment of other patients in the healthcare system.
